# MicroRNA-transcription factor network analysis reveals miRNAs cooperatively suppress RORA in oral squamous cell carcinoma

**DOI:** 10.1038/s41389-018-0089-8

**Published:** 2018-10-08

**Authors:** Xueqing Zheng, Kejing Wu, Shengjie Liao, Yuemei Pan, Yanan Sun, Xinming Chen, Yi Zhang, Shu Xia, Yaying Hu, Jiali Zhang

**Affiliations:** 1 The State Key Laboratory Breeding Base of Basic Science of Stomatology (Hubei_MOST) & Key Laboratory of Oral Biomedicine Ministry of Education, School & Hospital of Stomatology, Wuhan University, Wuhan, China; 2 Oral Histopathology Department, School and Hospital of Stomatology, Wuhan University, Wuhan, China; 3 Center for Genome Analysis, ABLife Inc, Wuhan, Hubei 430075 China; 4 Laboratory for Genome Regulation and Human Health, ABLife Inc, Wuhan, Hubei 430075 China

## Abstract

Oral squamous cell carcinoma (OSCC) represents over 90% of oral cancer incidence, while its mechanisms of tumorigenesis remain poorly characterized. In this study, we applied RNA-seq and microRNA-seq methodologies in four pairs of cancer and adjacent normal tissues to profile the contribution of miRNAs to tumorigenesis-altered functional pathways by constructing a comprehensive miRNA-mediated mRNA regulatory network. There were 213 differentially expressed (DE) miRNAs and 2172 DE mRNAs with the involvement of negative miRNA-mRNA interactions identified by at least two pairs of cancerous tissues. GO analysis revealed that the upregulated microRNAs significantly contributed to a global down-regulation of a number of transcription factors (TFs) in OSCC. Among the negative regulatory networks between the selected miRNAs (133) and TFs (167), circadian rhythm genes (*RORA*, *RORB*, *RORC*, and *CLOCK*) simultaneously regulated by multiple microRNAs were of particular interest. For instance, RORA transcript was predicted to be targeted by 25 co-upregulated miRNAs, of which, miR-503-5p, miR-450b-5p, miR-27a-3p, miR-181a-5p and miR-183-5p were further validated to directly target RORA, resulting in a stronger effect on RORA suppression together. In addition, we showed that the mRNA and protein expression levels of RORα were significantly decreased in most OSCC samples, associated with advanced clinical stage and poor prognosis. RORα significantly suppressed the proliferation of OSCC cells in vitro and in vivo. Attenuated RORα decreased p53 protein expression and suppressed p53 phosphorylation activity. Altogether, our results strongly suggest the importance of the role of miRNAs in regulating the activity of circadian rhythm-related TFs network during OSCC tumorigenesis, and provide further clues to understand the clinical link between circadian rhythm and cancer therapy.

## Introduction

Oral squamous cell carcinoma (OSCC), accounting for more than 90% of all oral cancers, is an aggressive malignant entity, characterized by local invasion, early lymph node metastasis and poor prognosis at an advanced stage^1^. Due to the phenotypic, aetiological, biological and clinical heterogeneity, individual molecular risk factors that have been investigated in OSCC have limited prognostic ability. In recent years, genetic studies, which have generated genomic annotation of molecular alterations in head and neck squamous carcinoma and other cancers, strongly support the idea that many of the current risk factors are likely coordinated at the biological pathway or network level rather than at an individual molecular level^2,3^. As an important type of gene regulatory networks, the miRNA-mediated transcriptome regulatory network, plays a vital role in the regulation of cell fate in many cancer types^4,5^. However, the role of this network remains unclear in OSCC.

MicroRNAs (miRNAs), approximately 22 nucleotide long endogenous non-coding RNAs, often recognize the 3′ UTR of mRNAs to perform the mRNA cleavage or translational repression^6–8^. It is well known that miRNAs exert key influences on fundamental biological processes such as cell death, cell proliferation, differentiation and apoptosis through fine tuning of their targets^9^. One of the important reasons is that a series of miRNAs are observed upregulated or downregulated coordinately generally in tumors^10–12^. They can target the same genes, and may possess cooperative oncogenic or carcinostatic functions by forming miRNA pairs^13,14^ or modulating transcription factor (TF) expression^15^. In addition, increasing numbers of studies have focused on the association between miRNAs and TFs in cancers. The dysregulation of miRNA-TF network always leads to the disorder of multiple signaling pathways and thus promotes malignant progression^15–17^. In OSCC, increasing abnormal expressed miRNAs and tumor suppressor TFs are recently discovered^18–20^. Despite these discoveries, the underlying molecular mechanism on how the miRNAs regulate the activity of transcription factors in OSCC remains poorly understood.

Retinoic acid receptor-related orphan receptor (RORα), a circadian rhythm gene and transcription factor^21^, is present in many tissues and cells including cerebellar Purkinje cells, liver, thymus, skeletal muscle, skin, lung, adipose tissue, and kidney^22^. It binds to ROR response elements, leading to various effects in key biological processes and pathological activities^23,24^. *RORA* maps to the middle of chromosome 15q22.2, a highly unstable region with frequent breaks in cancers, and acts as a tumor suppressor by inhibiting cancer proliferation, apoptosis and invasion^25–30^. The down-regulation of RORα has been observed in a variety of cancers including breast cancer^25,26^, colorectal cancer^27,28^ and prostate cancer^29^. Till now, the expression pattern and the potential function of RORα in OSCC development and progression are largely unknown.

In this study, we described a miRNAs-mediated TFs regulatory network using the deep sequencing and bioinformatics analysis by comparing the paired tumor and normal tissues. Importantly, in our study, we observed and described the cooperative effect of miRNAs on RORA. This study may provide new insights into the mechanisms of miRNAs-mediated regulatory network in OSCC.

## Results

### RNA-seq analysis revealed a number of differentially expressed genes in the cancer tissue compared to the normal tissue

**Fig. 1:**
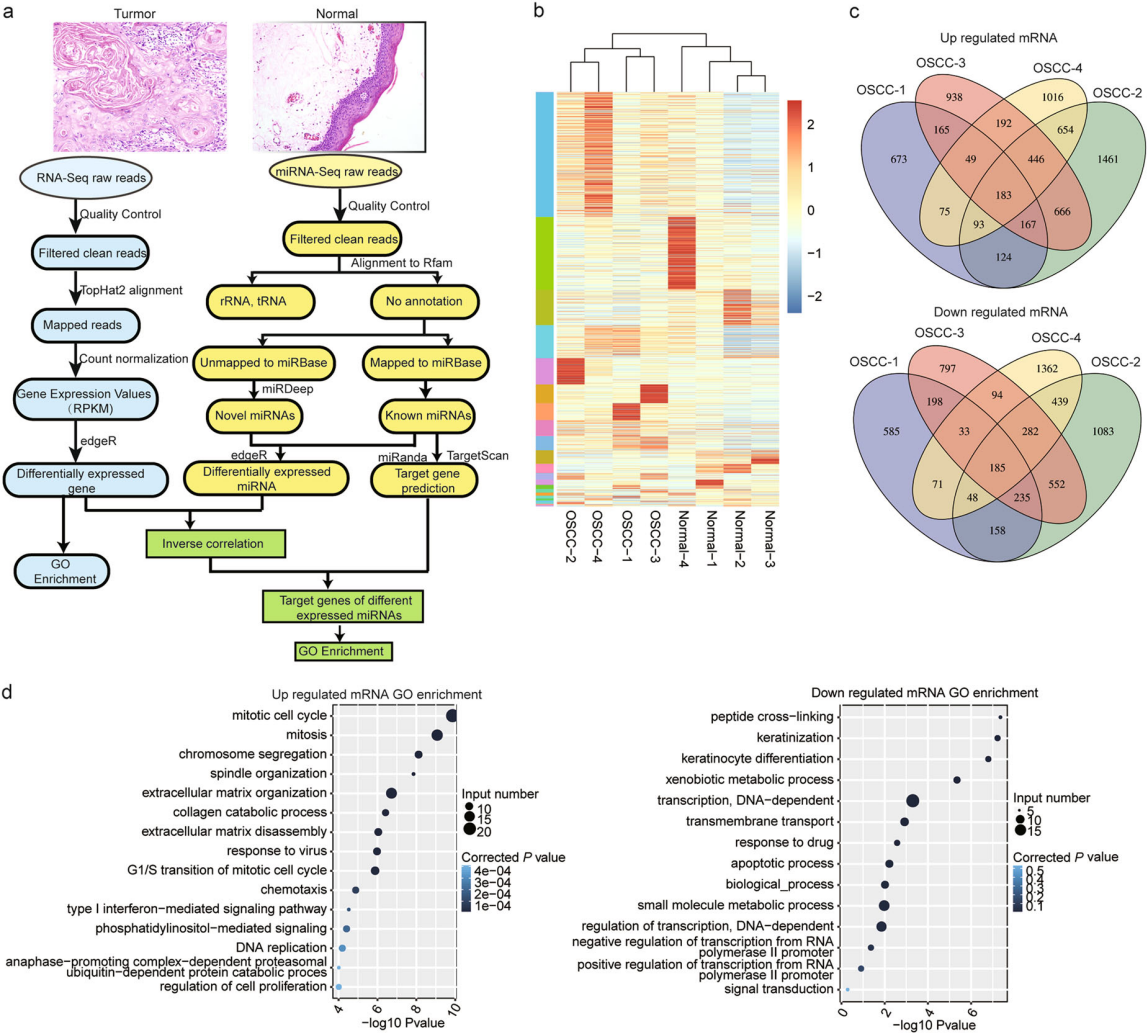
RNA-sequencing analysis showed the differentially expressed genes between OSCC tissues and normal tissues. **a** The flow chart described the data obtaining and analysis strategy of this study. The representative histological image of the OSCC tissue (up-left) and paired adjacent normal epithelia tissue (up-right) were also showed. Original magnification: x200. **b** Hierarchical clustering analysis showed that the differentially expressed mRNAs could discriminate the differences between cancerous tissues and para-carcinoma tissues. **c** Venn diagram analysis demonstrated the overlapping differentially up-regulated (up) or down-regulated (down) mRNAs between OSCC-Normal tissues (**d**) GO analysis of coordinately upregulated (left) or downregulated (right) genes in four OSCC tissues revealed the 15 most enriched pathways. The size of circle indicates the corresponding involved gene number; the color of corrected *p* value indicates the significance of the rich factor

To unveil how the transcriptional regulatory program was composed in OSCC and normal epithelium, paired cancer and adjacent normal epithelia specimens from four OSCC patients were collected for high-throughput RNA-sequencing. A flow chart describing the data obtaining and analysis strategy was applied in Fig. 1a. We obtained 18.5–35.8 million raw reads per sample. After removal of low-quality reads, between 16.0 and 28.3 million clean reads were retained for each RNA sample. Among them, total of 12.7–23.7 million reads (77.3–87.3% of total clean reads) were mapped to the human genome, from which 8.3 to 16.5 million (60.8–74.0% of total mapped reads) were uniquely mapped (Table S1). RNA-seq analysis showed that total of 33,375 genes expressed in at least one of the 8 samples, and between 21,977 and 24,584 expressed in individual samples. Among these, we detected 16,151 genes that had an RPKM ≥ 1 in any of the 8 samples, and between 11,075 and 12,808 genes detected from each sample, which ranged from 50.0 to 58.0% of the total expressed genes per sample (Table S2). A total of 11,788 genes were significantly differentially expressed in at least one pair of samples between tumor tissues and normal tissues. Subsequently, the expression values of all differentially expressed genes (DEGs) in each sample were extracted and bidirectional hierarchical clustering analysis was carried out. As shown in Fig. 1b, we observed that the DEGs could robustly discriminate the differences between cancerous tissues and para-carcinoma tissues. In addition, the overlapping up-regulated and down-regulated mRNAs between OSCC tissues were shown by Venn diagram (Fig. 1c). Among them, 183 mRNAs were coordinately upregulated and 185 mRNAs were coordinately downregulated in all of the cancerous tissues compared to normal tissues. To assess the biological function of differentially regulated genes, we performed gene ontology (GO) analysis. The results revealed that the consistently downregulated genes were mainly enriched under several GO terms, such as keratinization, transcription (DNA dependent, and regulation of transcription from RNA polymerase II promoter), apoptosis and signal transduction. On the other hand, the consistently upregulated genes were highly overrepresented in biological process related to mitotic cell cycle, spindle organization, extracellular matrix organization and disassembly (Fig. 1d).

### MiRNA-seq analysis revealed the differentially expressed miRNAs in cancer tissues compared to normal tissues

**Fig. 2:**
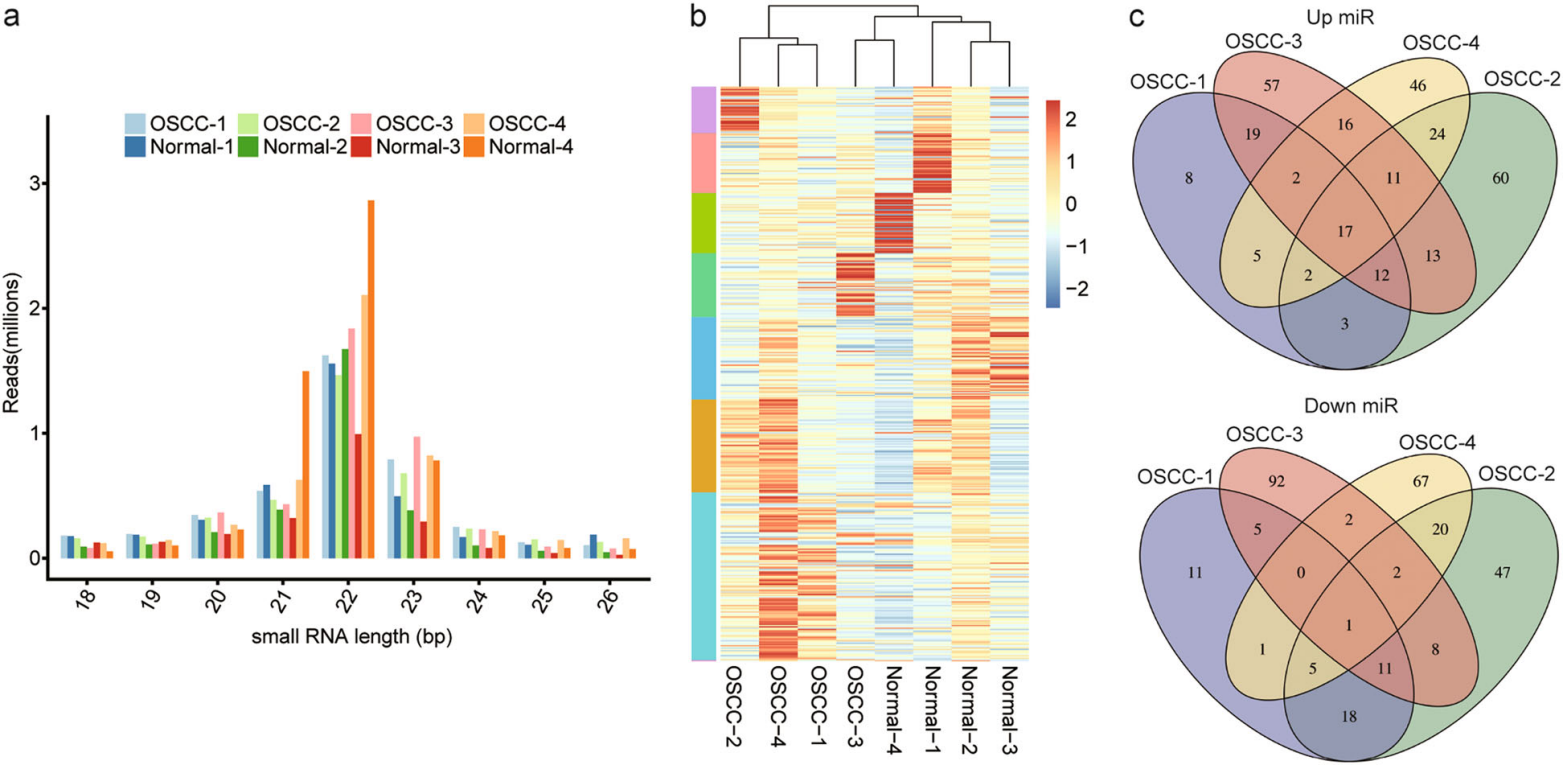
miRNA-sequencing analysis demonstrated the differentially expressed miRNAs between cancer tissues and normal tissues. **a** The distribution of small RNA length reads showed that the 21–23 nt small non-coding RNAs accounted for the majority of the reads. **b** Hierarchical clustering analysis showed that the differentially expressed miRNAs could almost discriminate the differences between cancerous tissues and para-carcinoma tissues. **c** Venn diagram analysis exhibited the overlapping differentially up-regulated (up) or down-regulated (down) miRNAs between OSCC tissues

In addition to RNA-seq data, we also extracted miRNAs from the same paired cancer and normal epithelium and performed miRNA-sequencing. The number of raw reads ranged from 4.9 to 7.6 million per sample. After discarding the low-quality reads, between 4.4–6.7 million clean reads represented 72.8–98.4% of the total number of reads initially obtained (Table S3). As shown in Figure 2a, the 21–23 nt small non-coding RNAs accounted for the majority of the reads, which was consistent with the size distribution of miRNA in human (Figure S1). Using Bowtie, 0.9–3.5 million reads mapped to known mature miRNAs, which ranged from 19.4 to 52.7% of the clean reads per sample. Clean reads that did not map to any known miRNAs were further analyzed to identify novel miRNAs, 153 novel miRNAs from the 8 libraries were identified using miRDeep algorithm. In total, there were 2617 miRNAs identified in at least a single sample, and between 1034 and 2067 expressed genes were detected in individual samples. Among these, we detected 946 miRNAs expressed above 1 TPM in any of the 4 paired OSCC and normal epithelia samples, and between 415 and 774 detected from each sample, which ranged from 34.1 to 46.7% of the total expressed miRNAs per sample (Table S4).

Of all expressed miRNAs, 458 in at least one pair of samples appeared to be significantly DE in tumor tissues compared to normal tissues. Hierarchical clustering of these miRNAs segregated tumors into clusters similar to those identified by expression profiling of differentially expressed gene. This suggested that miRNAs acted as key regulators of gene activity during the development of cancer (Fig. 2b). Additionally, the Venn diagram exhibited the specific number of differentially expressed miRNAs in every pair of OSCC-Normal tissue and the intersection region between samples (Fig. 2c). Among them, 17 miRNAs were coordinately upregulated and 1 miRNA was coordinately downregulated in all four cancerous tissues compared to the corresponding normal tissues. Unexpectedly, there were more coordinately upregulated miRNAs than coordinately downregulated miRNAs in OSCC tissues.

### The regulatory networks of miRNA-transcription factor were identified in OSCC after microRNA-mRNA sequencing

**Fig. 3:**
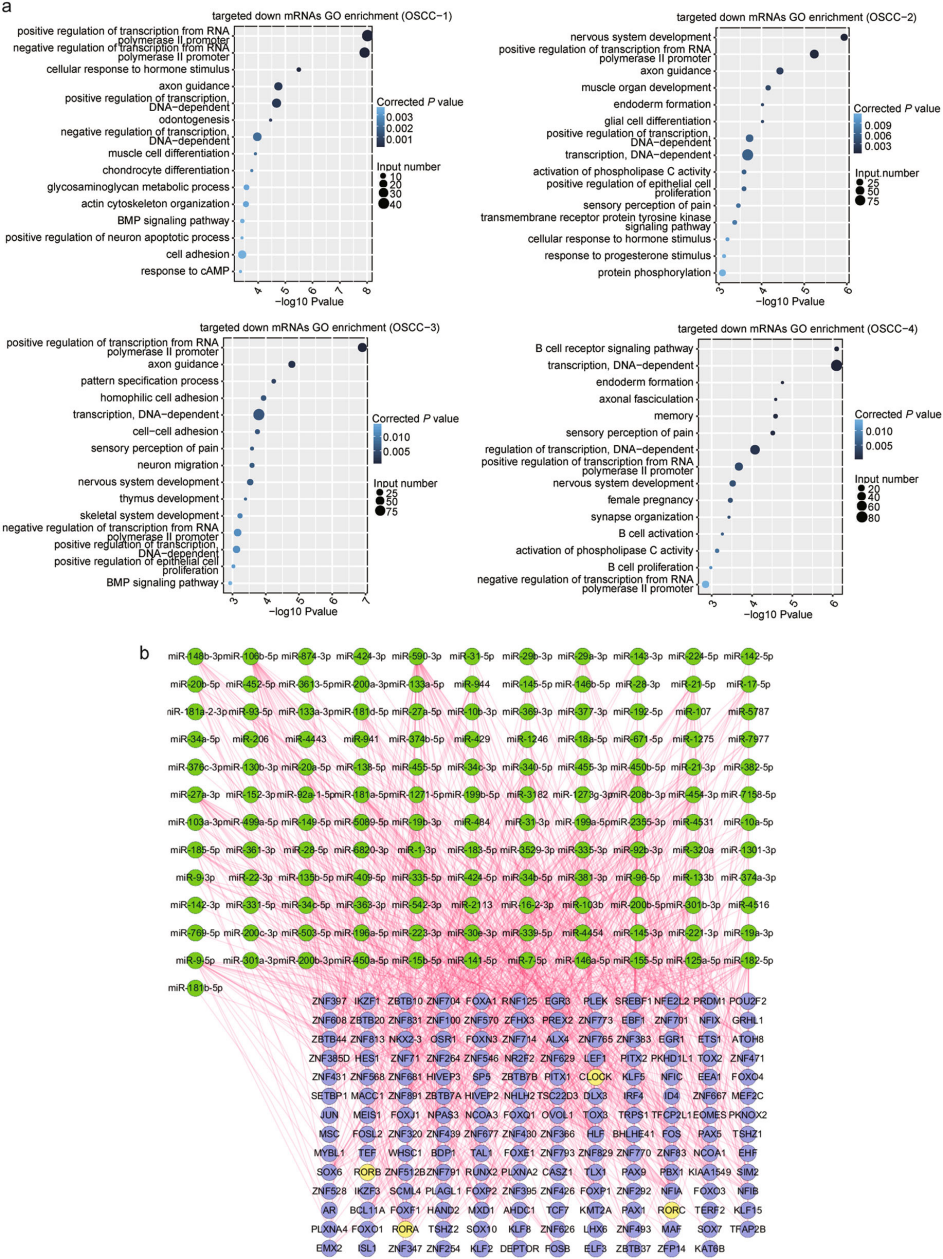
The predicted regulatory relationship between up-expressed miRNAs and targeted down-regulated mRNAs were identified. **a** GO analysis identified the top 15 enriched pathways based on the down-regulated mRNAs targeted by up-expressed miRNAs in four OSCC tissues, respectively. Transcription regulation related pathways were enriched and notably presented in every cancerous tissue. **b** The network displayed the negative regulatory relation between up-expressed miRNAs and the down-regulated TFs. Yellow circles highlight circadian clock genes

To understand the functional significance of regulated miRNAs in cancer, we performed an integrated analysis to build a miRNA-mRNA interaction network. In the first step, we used the miRanda and TargetScan to predict the target genes of miRNAs based on the complementary target sequence. The 3′ UTR sequences from all human transcripts were used as a custom target database, and 2588 known mature miRNAs in the miRBase 21 were used as a custom miRNA database. A total of 16,968 genes were predicted as potential targets of 2603 miRNAs. In the next step, the prediction was further filtered by negative correlation between miRNA and RNA expression. Predicted target lists of DE miRNAs were investigated in their respective DE mRNA list in order to delineate miRNA-mRNA interactions. Only inverse correlation between differentially expressed miRNAs and mRNAs identified by at least two or more cancerous tissues were retained for further analysis. We identified 8137 negative miRNA-mRNA interactions, involving 213 DE miRNAs and 2172 DE mRNAs, for further analysis. To elucidate the molecular function of predicted target genes, we performed GO analysis with down-regulated mRNAs targeted by upregulated miRNAs in four OSCC paired samples. According to the *p* value, the top 15 enriched pathways were identified and displayed in Fig. 3a. Noteworthy, although every cancerous tissue showed different pathway enrichment distribution, transcription regulation categories (positive or negative regulation of transcription from RNA polymerase II promoter pathways and DNA-dependent positive or negative regulation of transcription pathways) were enriched and notably presented in every cancerous tissue. The up-regulated miRNAs seemed to be closely related to the down-regulated TFs in OSCC tissues.

To further demonstrate the involvement of miRNA in regulating the expression of TFs, we selected the up-regulated miRNAs and the down-regulated TFs. A putative targeting network between miRNAs (133) and TFs (167) was shown (Fig. 3b). Importantly, the genes *RORA*, *RORB*, *RORC*, and *CLOCK* were simultaneously regulated by multiple miRNAs. Circadian rhythm related transcription factors play an essential role in the oncogenesis^31–33^, and these data suggest miRNAs may have a key role in their dysregulation.

Furthermore, we selected the down-expressed miRNAs and their target up-regulated mRNAs. The GO analysis was shown in Figure S2a and the regulatory network between down miRNAs and up TFs was exhibited in Figure S2b. In contrast, the GO term related to regulation of transcription was not enriched and the number of up regulated TFs was extremely low.

### RORA transcript was targeted by miR-503-5p, miR-450b-5p, miR-27a-3p, miR-181a-5p, and miR-183-5p

**Fig. 4:**
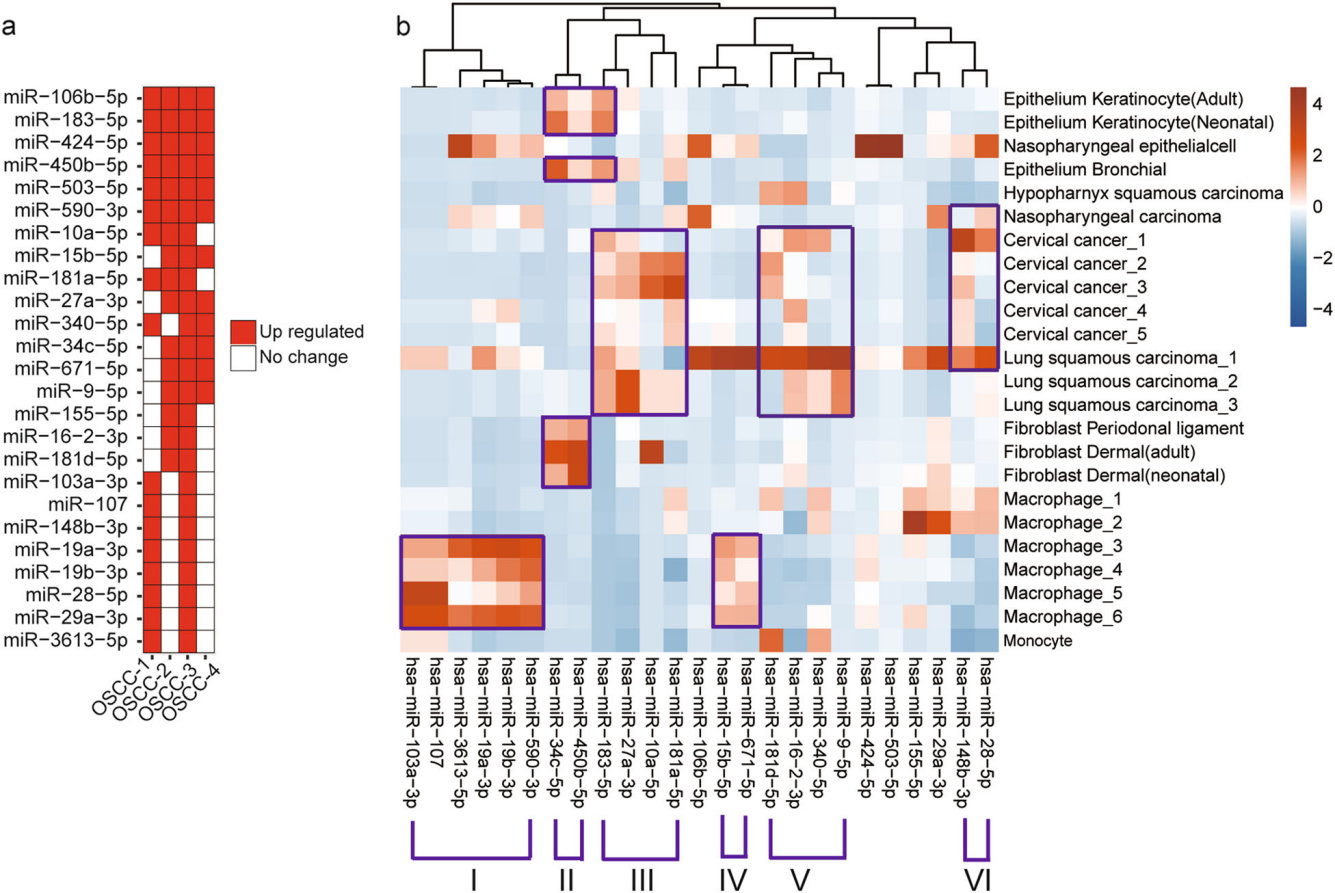
**a** Heatmap of the 25 miRNAs that potentially targeted RORA. The red square represents miRNA significantly up-expressed in OSCC tissues, and the white square means miRNA expression has no change in OSCC. **b** The heatmap showed 25 miRNAs abundances in 24 cell lines. The miRNA-seq data of these cell lines were obtained from McCall et al^39^. Columns and rows represent different miRNAs and cell types respectively

*RORA* has recently been identified as a novel circadian rhythm gene which plays an important role in tumor suppression by inhibiting the cell proliferation. According to the miRNA-TF regulatory network identified and described above, RORA was significantly suppressed in three of four OSCC tissues and potentially targeted by 25 onco-related miRNAs. Those 25 miRNAs were significantly up-regulated in at least 2 OSCC tissues, 8 of which were up-regulated in 3 OSCC tissues and 6 of which were up-regulated in 4 OSCC tissues (Fig. 4a).

Macrophage infiltration is common in tumors and plays a large role in mediating cancer-related inflammation^34–37^. Among the OSCC-upregulated miRNAs, we noticed that miR-590-3p had been reported to be highly expressed in macrophage and targeted the lipoprotein lipase gene transcript^38^. We suspected that some of the OSCC-upregulated miRNAs might be derived from the infiltrated macrophage. In light of this point, we analyzed the expression of the miRNAs upregulated in OSCC in different cell lines comprising macrophages, fibroblast, epithelium cells and tumors^39^. The results clearly demonstrated six interesting clusters (Fig. 4b). Among which two of them were highly expressed in most of macrophage cell lines. The major macrophage cluster harbored six of the 25 OSCC-upregulated miRNAs including miR-590-3p, miR-19b-3p, miR-19a-3p, miR-3613-5p, miR-107 and miR-103-3p, and the small cluster harbored miR-671-5p and miR-15b-5p. The two macrophage clusters might be associated with changes in macrophage infiltration. There was a small fibroblast-epithelium cluster including miR-34c-5p and miR-450-5p, and three cancer clusters. The larger cancer cluster included miR-106b-5p, miR-181a-5p, miR-10a-5p, miR-27a-3p, and miR-183-5p (Fig. 4b).

On the other hand, the six miRNAs that were consistently up-regulated in all OSCC samples included only one macrophage miRNA, miR-590-3p (Fig. 4a), suggesting that macrophage infiltration only had a partial contribution to the increased miRNA expression in OSCC. To determine whether decreased RORA expression in OSCC was caused by the onco-related miRNAs, we selected five miRNAs (miR-503-5p, miR-450b-5p, miR-27a-3p, miR-181a-5p, and miR-183-5p) which linked to tumor proliferation in a variety of cancers, to evaluate their regulation effects on RORA. Macrophage-associated miRNAs were excluded.

**Fig. 5:**
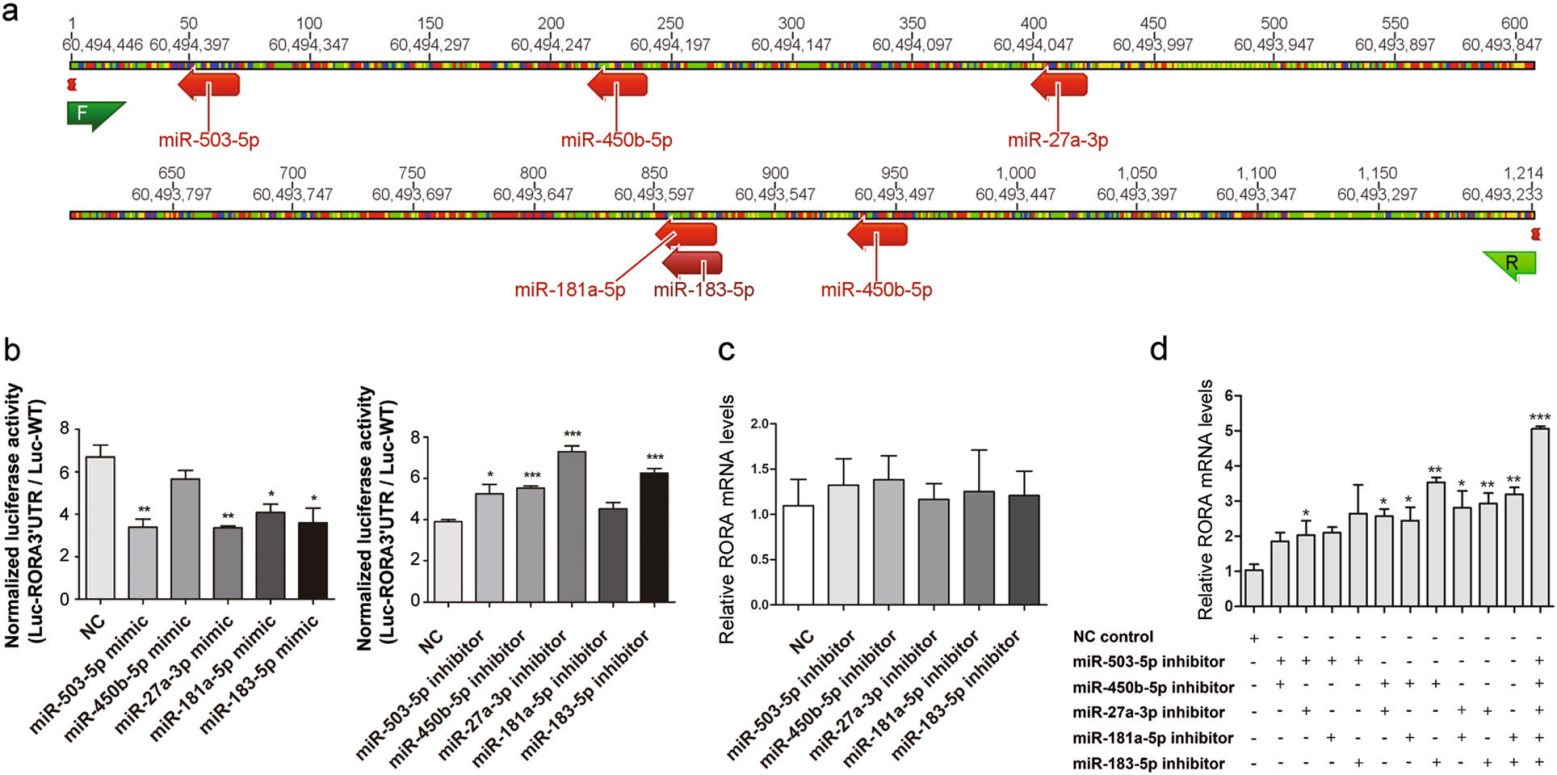
RORA was cooperatively suppressed by miR-503-5p, miR-450b-5p, miR-27a-3p, miR-181a-5p, and miR-183-5p. **a** RORA 3′ UTR sequence (bases 3009 to 4222 of the RORA transcript variant 1) enriched with predicted target sites of the 5 miRNAs was cloned into pSiCheck™-2 dual luciferase vector. **b** The five miRNA mimics reduced the luciferase reporter activity of pSiCheck™-2 RORA-3′ UTR (left). Inversely, the miRNA inhibitors increased the luciferase reporter activity (right). The final normalized luciferase activity was normalized by 3′ UTR/NC control. **c** The regulation effect of miRNAs on RORA mRNA expression. MiR-503-5p, miR-450b-5p, miR-27a-3p, miR-181a-5p, and miR-183-5p inhibitors (100 nM each miRNA, 60 h transfection) were transfected into HeLa cells, individually. **d** The cooperative regulation effect of miRNAs on RORA mRNA expression. The five miRNAs were transfected into HeLa cells in pairs with the final concentration of 400 nM (each 200 nM, 48 h transfection) or together with the final concentration of 500 nM (each 100 nM, 48 h transfection). MiR-NC was served as the negative control. *p < 0.05; **p < 0.01; ***p < 0.001

Binding sites for these 5 miRNAs were identified in the 3′ UTR of RORA using miRanda (v3.3a) and TargetScan (v6.0) and the corresponding region (bases 3009-4222 of the RORA1 transcript) was amplified for the dual-luciferase reporter assay(Fig. 5a). The luciferase reporter vectors inserted the cDNA of RORA 3′ UTR sequence or wild type vectors were co-transfected with miRNA mimics or NC scramble, respectively. Compared with the control group, the luciferase activities of miR-503-5p, miR-450b-5p, miR-27a-3p, miR-181a-5p, and miR-183-5p mimic group were reduced by approximately 49.4, 15.5, 49.8, 39.0, and 46.3%, respectively (Fig. 5b left). In the same way, RORA 3’ UTR-vectors or wild type vectors were also co-transfected with corresponding miRNA inhibitors or NC scramble. As a result, the miR-503-5p, miR-450b-5p, miR-27a-3p, miR-181a-5p, and miR-183-5p inhibitor group increased nearly 34.7, 41.7, 87.2, 16.1, and 60.4% luciferase activity, respectively (Fig. 5b right). Taken together, those five miRNAs presented different level of inhibitory effect on RORA mRNA translation by targeting RORA 3’ UTR sequence.

### RORA was cooperatively suppressed by miR-503-5p, miR-450b-5p, miR-27a-3p, miR-181a-5p, and miR-183-5p

To test the cooperative regulation effect of miRNAs on RORA expression, inhibitors of miR-503-5p, miR-450b-5p, miR-27a-3p, miR-181a-5p, and miR-183-5p were transfected into HeLa cells, individually, in pairs or together, and miR-NC served as negative control. According to the data from high-throughput sequencing, we detected the expression of three RORA isoforms (RORA 1, RORA 2, and RORA 4) in each pair of samples, while RORA 3 was not detected (Table S5). Compared to RORA 2 and RORA 4, RORA 1 exhibited higher RPKM values in samples and relatively significant fold change between normal and cancerous tissues. Therefore, RORA 1 was chosen for further research. The mRNA expression of RORA was consistently increased with individual miRNA inhibitors, however this effect was not necessarily significant (Fig. 5c). Additionally, when cells were transfected by miRNA inhibitors in pairs, there was an obviously increased RORA expression, compared with the control. Moreover, the 5 mixed miRNA inhibitors led to a much stronger up-regulation effect on RORA mRNA expression (about 4.9-fold) than the groups transfected with miRNA inhibitors in pairs (*p* < 0.001, Fig. 5d).

### The low expression of RORα was general in the OSCC tissues and associated with low survival rate

**Fig. 6:**
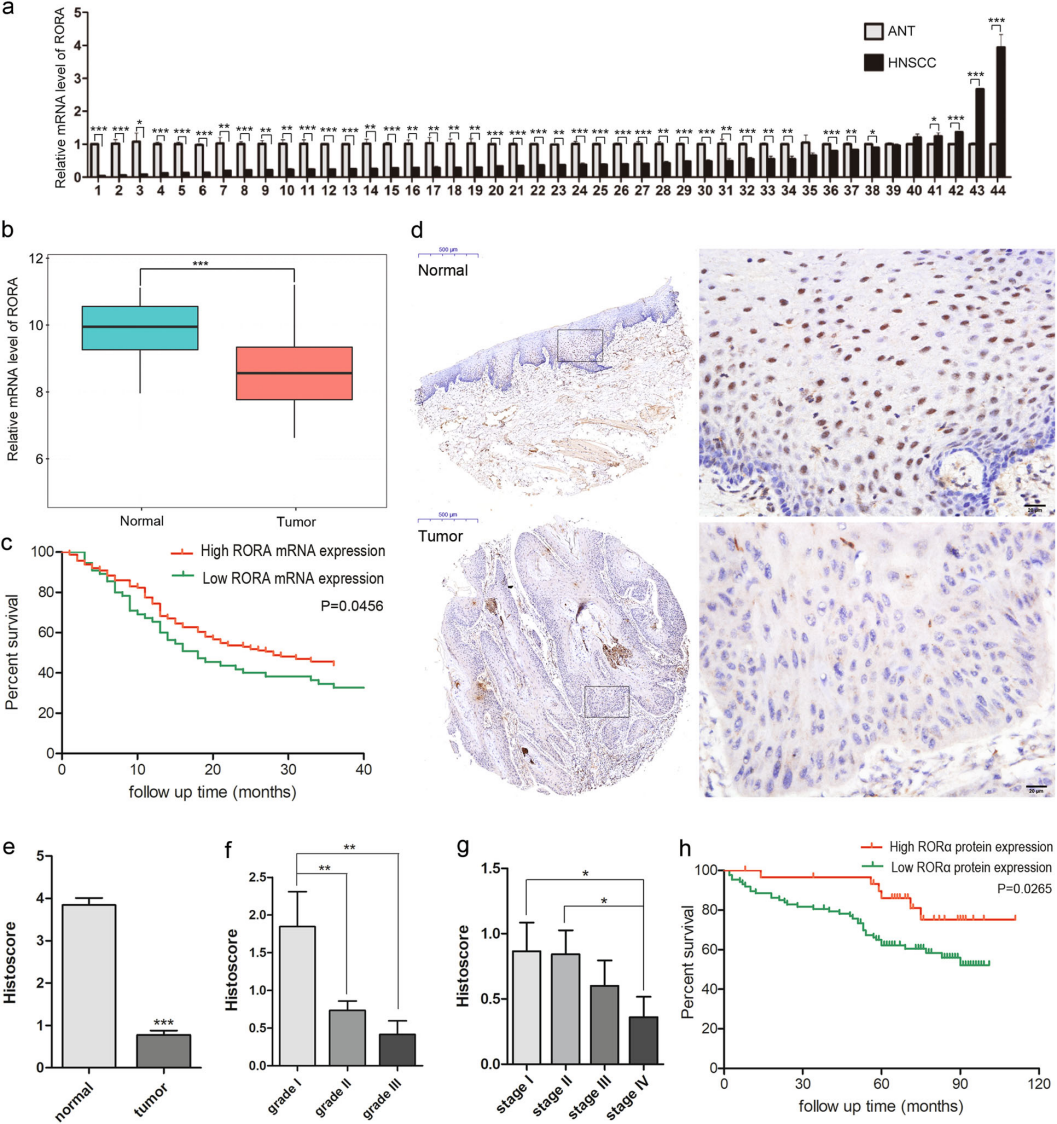
**a** Quantitative RT-PCR analysis showed the relative expressions of RORA were attenuated in 39 of 44 OSCC tumors, compared with the adjacent normal epithelial tissues (ANT). **b** Based on the RNA-seq data of 340 OSCC and 32 normal epithelial samples obtained from TCGA database, RORA exhibited significantly lower mRNA abundance in the cancer samples than that in normal epithelial. **c** The Kaplan–Meier survival analysis showed that patients with relatively low RORA mRNA expression were correlated with poor 3-year overall survival (*p* *=* 0.0456). **d** Representative images of RORα staining in adjacent normal epithelium and OSCC tissues. Corresponding scale bar was shown in the images. **e** The histoscore in OSCC tissues was lower than that in the adjacent normal epithelial. **f** Lower protein expression of RORα was significantly correlated with more advanced histological grade. **g** RORα protein expression presented a decreasing trend in clinical stage I, stage II, stage III and stage IV. **h** The Kaplan-Meier survival analysis showed that patients with low RORα protein expression were correlated with unfavorable 5-year overall survival (*p* = 0.0205). *p < 0.05; **p < 0.01; ***p < 0.001

The large number of miRNAs involved in controlling the expression of RORA suggested its importance in tumorigenesis. In order to assess this hypothesis, the mRNA levels of RORA in the tumor and normal epithelial tissues from 44 OSCC patients were studied using the quantitative real-time PCR. We showed that RORA transcription was significantly decreased in most OSCC samples (39 of 44, 88.6%), compared with the adjacent normal epithelia (Fig. 6a). The result was consistent with our mRNA sequencing data which demonstrated that 3 of 4 OSCC samples showed a reduction of RORA transcript.

To further explore RORA transcript abundance in OSCC, we analyzed RORA levels in the TCGA RNA-seq data from 340 OSCC and 32 normal epithelial samples. Consistent with our data, RORA exhibited significantly lower expression in the cancer samples from TCGA (*p* < 0.001, Fig. 6b). Using the mRNA expression and corresponding clinical data, we examined 3-year overall survival for 219 OSCC patients where samples with RORA abundance lower than the first quartile value were classified as low expression. As shown, relatively low RORA abundance was associated with poor survival (*p* = 0.0456, Fig. 6c).

To further investigate the association between RORα protein and the clinicopathological characteristics of OSCC patients, the protein level and expression location of RORα were detected in 125 OSCC tissues and paired adjacent epithelia. In the adjacent normal epithelial, RORα localization was well-defined, exclusively within cell nuclei with strong staining, while in OSCC cells, it mainly showed faint or negative nuclear expression accompanied with slight cytoplasm staining (Fig. 6d). Statistical analysis revealed that the level of RORα protein in OSCC cells is significant lower than that in adjacent normal epithelial cells (*p* *<* 0.001, Fig. 6e).

Next, we analyzed the association between RORα expression and histological grade, clinical stage, and patient outcomes. Attenuated nuclear RORα abundance was detected in tumors with a poorly-differentiated histology and advanced clinical stage (*p* < 0.01, Figs. 6f, g). In total, 125 OSCC patients were followed up until death or more than 5 years (range, 60–111 months). During the follow-up period, 36 patients succumbed to the disease within 60 months, 6 patients succumbed to the disease more than 60 months, 76 patients were alive, and 7 patients were lost. The samples with the staining score ≥ 1.07 were classified as high RORα protein expression, otherwise as low expression. The Kaplan-Meier survival analysis and the log-rank test showed that patients with a low level of RORα were significantly associated with unfavorable 5-year survival (*p* = 0.0265, Fig. 6h).

### The five OSCC onco-miRNAs expression was widely upregulated in the tumor tissues of OSCC patients

**Fig. 7:**
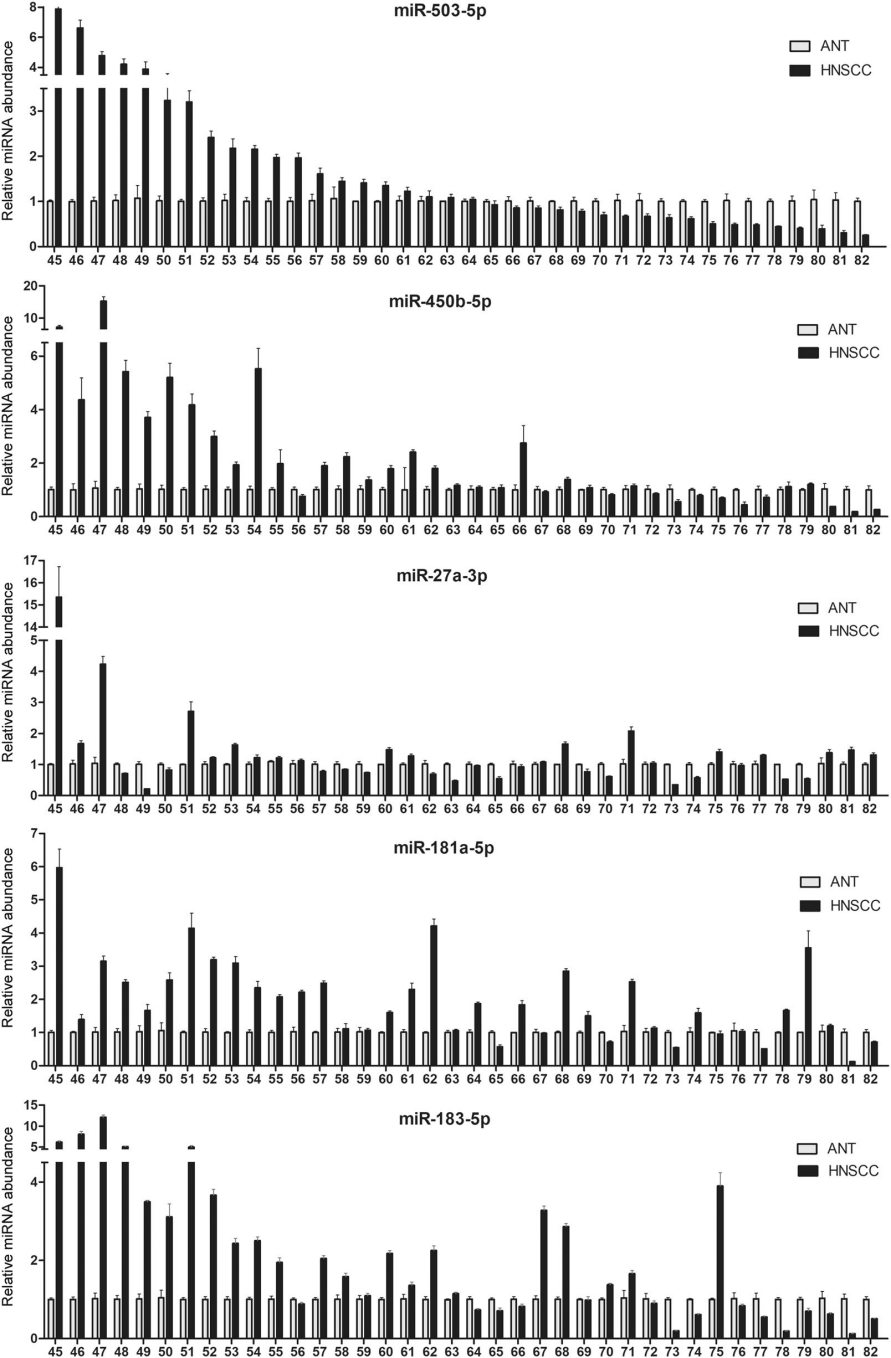
Quantitative RT-PCR analysis showed that miR-503-5p, miR-450b-5p, miR-27a-3p, miR-181a-5p, and miR-183-5p were upregulated in 52.6, 68.4, 52.6, 78.9, and 60.5% of the OSCC tissues, respectively, compared with the adjacent normal tissues (ANT)

To explore whether upregulation of miR-503-5p, miR-450b-5p, miR-27a-3p, miR-181a-5p and miR-183-5p was general in OSCC, their expression level was detected in 38 paired adjacent epithelia and OSCC tissues. All five miRNAs examined were upregulated in the majority of samples tested: miR-503-5p in 52.6%; miR-450b-5p in 68.4%; miR-27a-3p in 52.6%; miR-181a-5p in 78.9%, and; miR-183-5p in 60.5% (Fig. 7). These data were in good accordance with our miRNA-seq results showing that these miRNAs were increased in oral cancers, compared with the adjacent normal epithelia.

### RORα reduced tumor cell proliferation in vitro

To elucidate the function of suppressed RORα expression on tumorigenesis, OSCC cell lines stably inhibiting RORα expression were generated by transfection of GV-248-RORα-sh1/-sh2/-sh3 lentiviral vectors into Cal-27 cells.

**Fig. 8:**
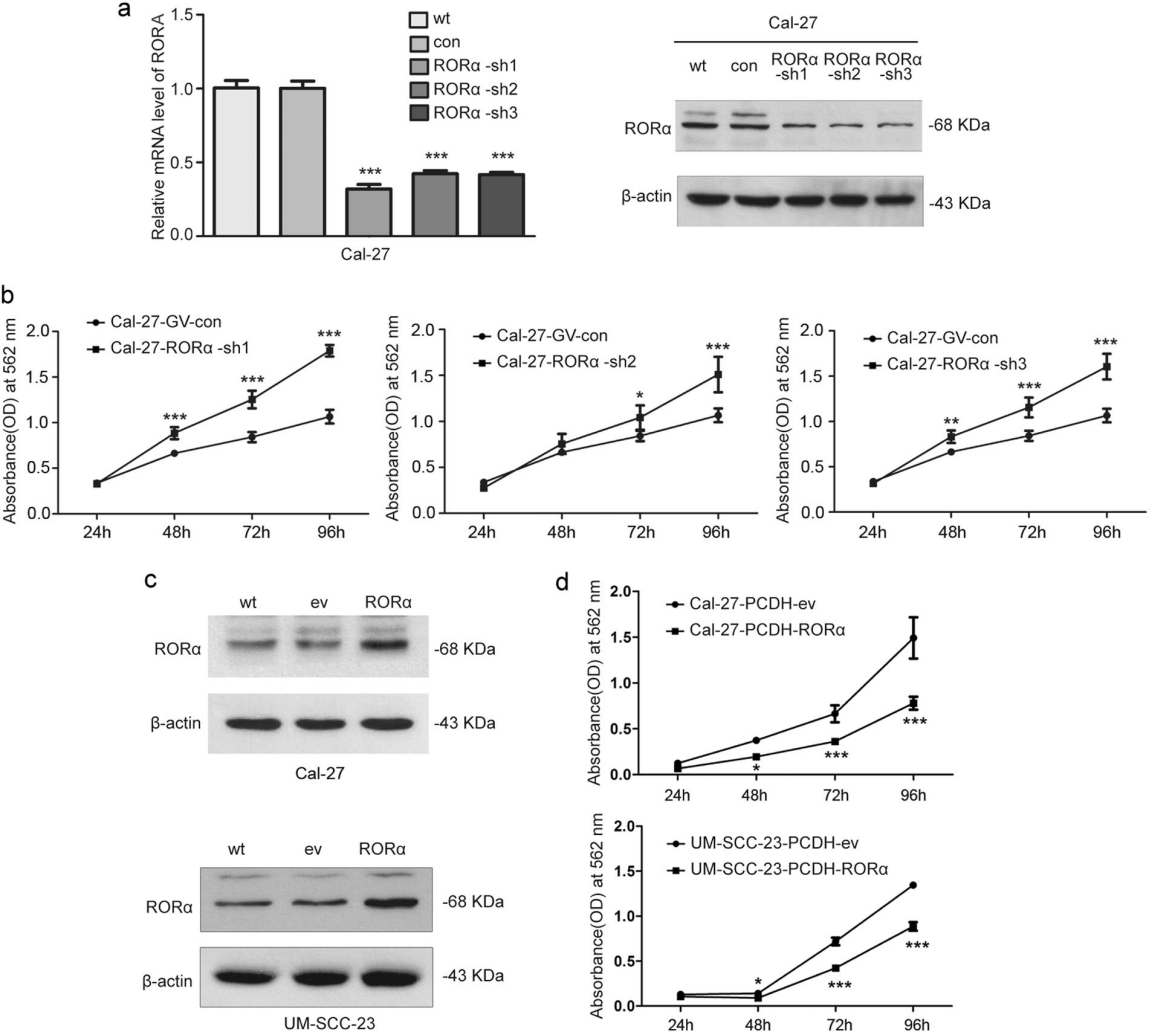
**a** Inhibiting RORα expression levels in Cal-27 cells using three constructed sh-lentiviral vectors GV-248-RORα-sh1, 2 and 3 by qRT-PCR (left) and western blot (right). **b** CCK-8 assays were performed to evaluate cell proliferation in Cal-27-RORα-sh1, 2, and 3 cells respectively, compared to control group. **c** Stable RORα overexpression in Cal-27 cells (up) and UM-SCC-23 cells (down) was assessed by western blotting. **d** CCK-8 assays were performed to evaluate cell proliferation in Cal-27-RORα cells (up) and UM-SCC-23-RORα cells (down), compared with control cells. *p < 0.05; **p < 0.01; *** *p* *<* 0.001

The mRNA and protein abundance of RORα was reduced (Fig. 8a) and in Cal-27 cells RORA targeting shRNAs significantly accelerated the cell growth rate by 1.68-fold (sh1), 1.42-fold (sh2) and 1.51-fold (sh3) 4 days after using CCK-8 assay reagent (*p* < 0.01, Fig. 8b).

We then determined whether the restoration of RORα expression could produce the opposite effect on cell growth. Therefore, *RORA* gene was stably over-expressed in Cal-27 and UM-SCC-23 cells using pCDH-EF1-puro lentiviral vector encoding RORA CDS sequence. Then, stable clones of Cal-27-RORα and UM-SCC-23-RORα were generated and caused a significant increase of RORα’s mRNA and protein expression level (Fig. 8c). The ectopic RORα expression resulted in 48% decrease in Cal-27 cell growth rate, and 34% decrease in UM-SCC-23, compared with the cells transfected with control vector four day after using CCK-8 assay reagent (*p* < 0.01, Fig. 8d).

### Restoring RORα expression reduced tumor growth in vivo

**Fig. 9:**
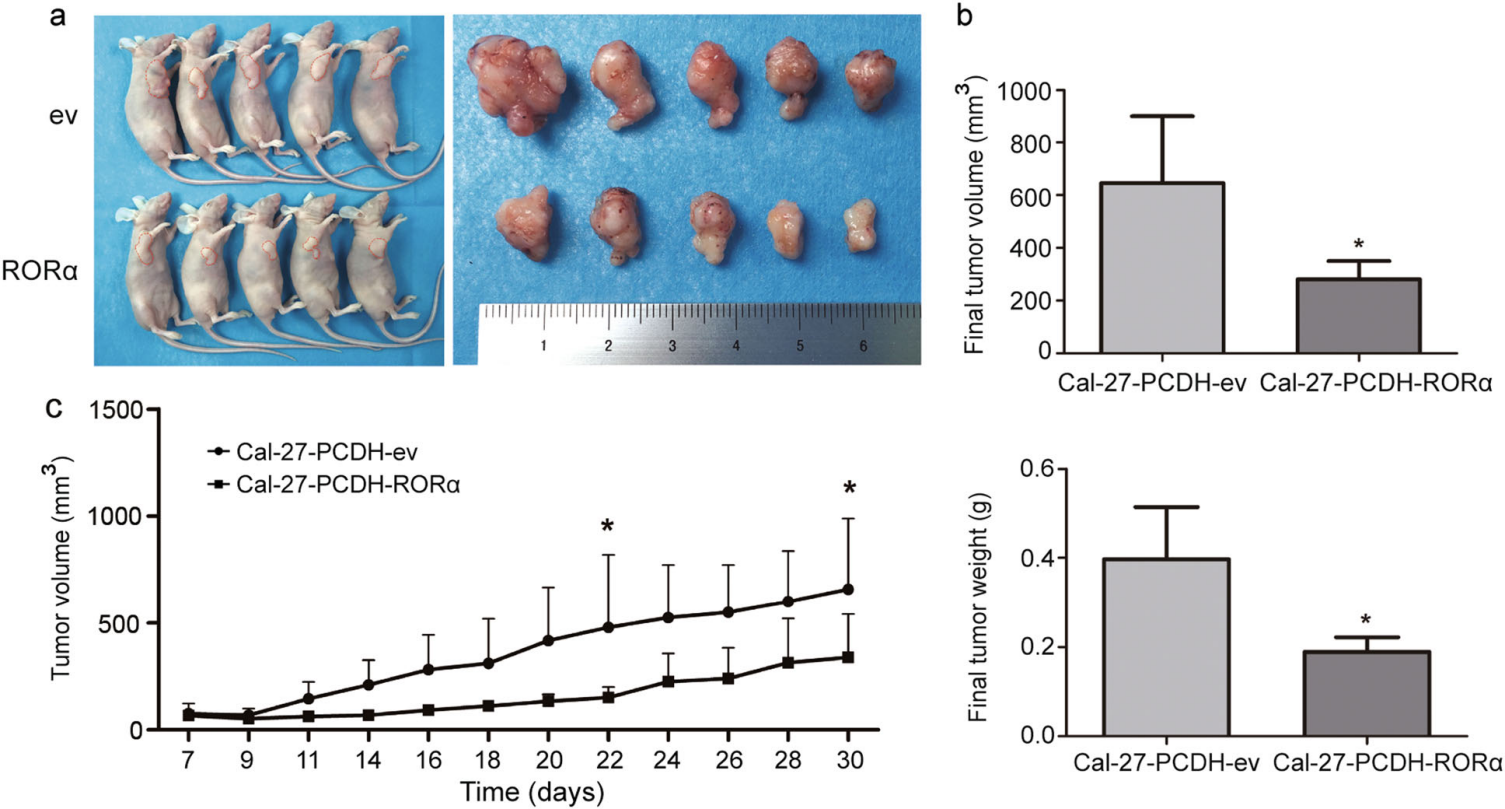
**The enforced expression of RORα suppressed tumor growth in vivo.** **a** Representative images of the sacrificed BALB/c nude mice bearing OSCC xenografts (left) and the isolated tumors (right). The red dotted circle showed the area of xenograft tumor. **b** The tumor volumes (up) and the tumor weights (down) of OSCC xenografts when mice were scarified. *n* = 5. **c** The growth curves of xenograft tumors after the subcutaneous injection of indicated cells. *n* = 5. *p < 0.05; **p < 0.01; ***p *<* 0.001

To identify the effect of enforced RORα on inhibiting tumor growth in vivo, Cal-27 cells overexpressing RORα or transfected with the control vector were injected subcutaneously into the left axilla of nude mice. On the 7th day, the tumor nodes were palpable. And 30 days later, Cal-27-RORα cells generated significantly smaller and lighter tumor nodes than control cells (Fig. 9a). Statistically, in comparison with the control group, the final volumes and weights of Cal-27-RORα xenografts were dramatically decreased about 56.4 and 52.4%, respectively (*p* < 0.05, Fig. 9b). The relative tumor growth inhibition rate in Cal-27-RORα xenograft model compared with Cal-27-ev was shown in Fig. 9c.

### RORα positively regulated p53 expression and phosphorylation

**Fig. 10:**
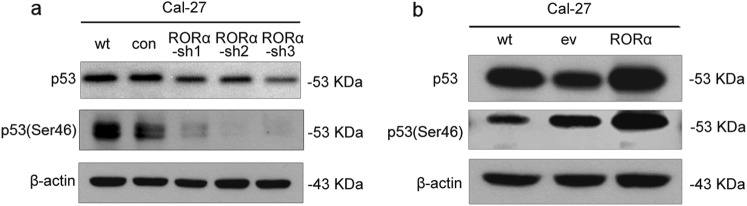
The protein levels of total and phosphorylated p53 in Cal-27 with RORα knockdown (**a**) and overexpression (**b**) were detected by western blot

RORα has been reported to form a regulatory loop with the tumor suppressor p53. We then explored whether RORα acted as a support suppressor in OSCC with an effect on p53. Here, we found that attenuated RORα expression not only decreased p53 protein level, but also inhibited p53 phosphorylation at its N-terminal serine 46 (Ser46) (Fig. 10a). Inversely, forced RORα expression led to increased protein levels of p53 and p53 Ser46 (Fig. 10b).

## Discussion

It is well-known that miRNA-TF mediated transcriptional regulation plays a vital role in the development and progression of many solid cancers^40^. In this study, we have explored the power of miRNA-TF regulation in driving the regression of OSCC. The paired RNA-seq and miRNA-seq analysis have revealed an important role of miRNA upregulation in driving the globally down-regulated TFs. MiRNA-TF network analysis led to the discovery of four circadian rhythm genes including *RORA* whose transcript was targeted by up to 25 expressed miRNAs. We have experimentally validated a group of oncomiRs (miR-503-5p, miR-450b-5p, miR-27a-3p, miR-181a-5p and miR-183-5p) in repressing the expression of RORA, which is proven to be a major tumor suppressor by clinic sample analysis, as well as by cell proliferation and nude mice assays. We further showed that RORα may partially exert its tumor suppressor activity by promoting p53 expression.

In this study, we used RNA-seq approach to profile consistently up- or down- regulated mRNA genes discriminating OSCC tissues from normal tissues. In accordance with previous research, the functional pathways related to tumor growth and invasion such as “mitotic cell cycle and extracellular matrix organization” were notably over represented in the OSCC-upregulated genes^41^. In contrast, genes enriched in pathways controlling normal epithelial biological functions including keratinocyte differentiation, xenobiotic metabolic process, transcription, apoptotic process, etc. were predominantly subdued. The dysregulated genes and pathways might demonstrate the underlying molecular mechanisms during malignant transformation and progression of OSCC.

Changes to miRNA expression in OSCC have been shown to drive transcriptional dysregulation for genes critical in tumorigenesis^18,19,42,43^. However, the molecular interplay between miRNA and mRNA has not been comprehensively profiled. Our findings are consistent with the studies in the last few years, showing that the miRNA-TF regulatory network plays a key role in regulating signaling and transcriptional pathways in various cancers, including glioblastoma multiforme^15^, ovarian cancer^16^ and malignant germ cell tumor^17^. Recent studies have discovered that although miRNAs are reported to only have minor influence on the target gene expression by fine tuning their targets, they have profound effect on cell-fate determination through targeting TFs and forming the miRNA-controlled feed forward loop^15,40^. The dysregulation of transcription factors which are points of convergence for multiple signaling pathways contributes to the pathogenesis of many cancers^44,45^. In this study, miRNA-TF regulatory network analysis has revealed a number of key transcription factors repressed by multiple miRNAs, including four circadian rhythm genes (three members of retinoic acid receptor-related orphan receptor family (*RORA*, *RORB*, and *RORC*) and *CLOCK*).

Among these TFs, RORA is further demonstrated to be widely suppressed in OSCC samples, and represses cell proliferation in vitro and tumor growth in nude mice. These results strongly suggest that RORα is a master tumor suppressor in OSCC progression. It has been reported that RORα is involved in many physiological and pathological processes. It inhibits breast tumor invasion^25,26^ and cell proliferation^27,28^, as well as it increases the cell apoptosis^30^. As an important transcriptional cofactor, RORα plays a crucial role in activating or inactivating some key tumor suppressor genes or oncogenes. For example, RORα can stabilize p53 and activate p53 transcription in a HAUSP/Usp7-dependent manner^30^, promote Bmal1 transcription^21^, inhibit the Wnt/β-catenin signaling pathway^27,28^, and decrease the expression of 5-LOX^29^. Our results demonstrate that RORα promotes p53 expression and phosphorylation, indicating it may exert its tumor suppressor function partially through the interplay with p53.

The loss of RORα expression is a decisive factor in breast cancer^25,26^, colorectal cancer^27,28^ and prostate cancer^29^. However, the mechanism of RORα down-regulation in cancers remains unclear. Previous studies have shown that RORA could be epigenetically silenced by aberrant DNA methylation in gastric cancer^46,47^ or by miR-17~92 in T follicular helper cells^48^. Here, we demonstrate a novel mechanism of down-regulation of RORA in OSCC, where up to 25 miRNAs target RORA 3′ UTR sequence and cooperatively suppress RORA transcript abundance, suggesting a possible mechanism of miRNA regulated RORA reduction in OSCC.

OSCC is known to have a high degree of inflammation^49^. Besides the cancer cells, the inflammation microenvironment of OSCC contains large amount of non-neoplastic stromal cells, including macrophage, fibroblast, etc. Among the up-regulated 25 miRNAs detected in our research, several of them (e.g., miR-590-3p and miR-103a) are reported actively expressed in cancer-related macrophages, promoting tumor growth and metastasis^50^. Therefore, we further explore the 25 miRNAs abundances in the cell lines associated with cancer-related inflammation microenvironment. According to the data released by McCall et al^39^, our results demonstrate that a smaller portion of miRNAs up-regulated in macrophage and a larger portion of miRNAs up-regulated in cancer cells, suggesting macrophage could partially contribute to the increased miRNA expression in cancer. These results are consistent with a model of intimate macrophage-cancer cell interactions in OSCC, and may contribute to the OSCC-upregulated miRNAs^51,52^. Currently miRNA-seq data from isolated OSCC and OSCC stromal cells is limited, therefore, the miRNA changes in different cell types are not fully understood. Further research and a greater number of cell line models may help to clarify the miRNA changes driven by tumor-environment crosstalk.

In our further experiments of testing miRNAs capability in targeting RORA 3′ UTR, we have experimentally validated five miRNAs including miR-503-5p^53^, miR-450b-5p^54^, miR-27a-3p^55–57^, miR-181a-5p^58^ and miR-183-5p^59,60^ can directly target RORA 3′ UTR and moderately suppress RORA expression. Their effect of suppression increases by cooperation. Thus, we speculate that up-regulation of a group of miRNAs cooperatively targets RORA 3′ UTR and thereby synergistically facilitates an effective gene repression. This hypothesis is consistent with the recent findings that a set of miRNAs up-regulated or down-regulated in cancers have a cluster of overlapping target genes, and therefore are able to regulate genes in a pleiotropic and coefficient pattern^13,14,61^.

Beyond a role in controlling circadian rhythm genes, the miRNA-TF network also reveals miRNA effects on epithelial-mesenchymal plasticity/phenotype switching. In this study, a group of EMT related transcription factors are identified up-/down- regulated in OSCC accompanied with several identified miRNAs. Changes of some genes are concordant with the phenotypic changes of the tumor cells (such as *SOX4*, *SOX12*, *SOX9*, *E2F1*, *E2F3*, *E2F7*, *E2F6*, *FOSL1*, *SNAI2*, and *HMGA2*, see in Figure S2). Those EMT-related genes are detected differentially up-regulated in tumor tissues and have several identified down-expressed miRNAs such as miR-204-5p, miR-200a-3p, Let-7, etc^62–65^. These miRNA-TF changes demonstrate the dysregulation of miRNAs plays an essential role in OSCC carcinoma-associated EMT. On the other hand, there are a small cluster of EMT related transcription factors, whose changes are inversely correlated with the tumor EMT phenotype. For example, genes including *LEF1*, *OVOL1*, *FOS*, *FOSL2*, *EHF*, and *SOX10*, are down-regulated (see in Fig. 3b), several of which accompanied with identified miRNAs up-regulation (including miR-181a-5p-5p, miR-29b-3p and miR-30e^66–68^). The unexpected changes of these genes suggest complicated mechanisms on TF-miR interaction, which might provide a feedback or feedforward loop and cross-gene regulation consequently^69^. Further research is required to uncover the potential molecular mechanisms of TF-miRNA interaction on tumor EMT switching in OSCC.

To our current knowledge, the mechanism by which way miRNAs are involved in tumor suppression is extremely complex. For the first time, we discovered a miRNAs-mediated TFs network, which led to a discovery of a master tumor suppressor of OSCC progression, RORA, whose expression was cooperatively controlled by a group of regulated miRNAs. Moreover, circadian rhythm is reported to be disturbed in breast cancer patients, and affects cancer chemotherapy^70,71^. As a regulator involving in circadian rhythm, the finding of RORα as a critical OSCC suppressor and potentially controlled by a large number of miRNAs might provide clues to further understand OSCC development and progression and develop anti-OSCC therapies, as well as to understand the clinical link between circadian rhythm and cancer.

## Methods and materials

### Clinical sample collection

Four pairs of fresh primary OSCC tissues and the matched adjacent normal epithelial tissues collected in 2012 were used to perform RNA-seq and miRNA-seq. Forty-four pairs of fresh primary OSCC tissues and the matched adjacent normal epithelial tissues collected from 2012 to 2013 were used to detect RORA expression. Thirty-seven pairs of fresh primary OSCC tissues and the matched adjacent normal epithelial tissues collected from 2014 to 2017 were used to detect the miRNAs expression.

The OSCC tissue microarrays were obtained from 125 OSCC paraffin embedded samples during the period between 2002 and 2012. All the adjacent normal epithelium samples were obtained by local excision. The histological types and tumor grades were analyzed by two pathologists. There were 250 spots in all from 125 patients: for each patient, two spots were selected, respectively, from normal adjacent epithelium and cancer area. The 250 spots were then spread evenly on 6 slides. The diameter of each spot was 2 mm. Tissue microarrays were produced by Outdo Biotech (Shanghai, China) after the design. The clinical information related to the samples was listed in Table S6.

All the samples were collected from patients of OSCC undergoing primary surgery in Stomatology Hospital of Wuhan University. The Ethics Committee of Wuhan University has approved the examination of patient samples.

We selected 340 OSCC and 32 normal epithelial samples downloaded from TCGA to compare the RORA mRNA abundance (https://xena.ucsc.edu/welcome-to-ucsc-xena/). Furthermore, 219 OSCC patients with over 3-year follow-up period were selected to perform overall survival analysis.

### RNA-seq and miRNA-seq

Total RNA from the tissue specimens was extracted using TRIzol Reagent (Ambion) following the manufacturer’s instructions. RNA-seq libraries were prepared with RNA-seq Library Preparation Kit (Gnomegen) following the manufacturer’s instructions and sequenced on the Illumina Hiseq 2000 (101 bp paired-end) and Illumina GAIIx (73 bp paired-end). Small RNA cDNA libraries were generated with Balancer NGS Library Preparation Kit for small/miRNA (Gnomegen) and applied to the Illumina GAIIx (Illumina, San Diego, CA, USA) 73 nt single-end sequencing.

### RNA-seq data processing

For RNA-seq data, adaptors and low quality bases were trimmed from raw sequencing reads using FASTX-Toolkit (Version 0.0.13), and reads less than 16nt were discarded. Quality control checks were performed by FastQC. After data filtering and quality control, reads were aligned to the reference genome GRCh38 by TopHat2 (v2.1.1), allowing up to 4 mismatches. Reads only unambiguously aligned to unique position were preserved to calculate reads number and RPKM (reads per kilobase and per million) value for each gene.

### MiRNA-seq data processing

After the completion of sequencing, raw reads were processed by FASTX-Toolkit (Version 0.0.13) to obtain reliable clean reads. During this procedure, adaptor sequences, low quality tags and sequences shorter than 16nt were removed. The obtained high-quality clean reads were searched against the Rfam database (version 12.0) using Bowtie allowing one mismatch. The matches to rRNAs and tRNAs were excluded. The remaining reads were aligned against the miRBase (v21) using Bowtie allowing one mismatch. Unaligned sequences from the 8 libraries were pooled together to identify novel miRNAs using the software package miRDeep (v2.0.0.5) with default parameters. To investigate the expression profiles of miRNAs, the frequency of miRNA counts were normalized to TPM (tags per million) using the following formula: normalized expression = actual miR read count / total clean read count × 10^6^.

### MiRNA target prediction

The potential miRNA targets were identified using miRanda (v3.3a) and TargetScan (v6.0) with default settings. All predicted target genes were evaluated by scoring system, and the sequences were considered to be miRNA targets only if miranda score was more than 500 or targetscan context + score was less than 0. To reduce the false identification rate, the gene expression profile of putative targets was required to be negatively correlated with the profile of miRNAs. For each paired samples, we identified the set of differentially expressed target genes and their corresponding miRNAs significantly changing in the opposite way. The mRNA-miRNA pairs whose negatively correlated relationship was detected in at least two paired samples were retained further to plot network by Cytoscape^72^.

### Differentially expressed genes and miRNAs

Differentially expressed genes and miRNAs between the paired groups were analyzed by using edgeR^73^ in R packages. The genes/miRNAs were retained for further analysis if their expression reached count-per-million levels above 1 in at least 1 sample. For each gene, significance *p*-value and FDR were obtained based on the model of negative binomial distribution. Fold changes of gene expression were also estimated within the edgeR statistical package. The criterion for DEG and DEmiR has been set as |fold change| > 1.5 and FDR < 0.05.

### GO enrichment

Pathway analysis was performed using KOBAS (version 2.0)^74^, based on the Gene Ontology (GO) database^75^. The KOBAS default method “h” based on the Wallenius non-central hypergeometric distribution was used to evaluate whether a specific pathway was statistically significant above the random chance. FDR (Corrected *p*-value) was calculated on the results of the pathway analysis using Benjamini-Hochberg method by KOBAS.

### Cell culture

The Cal-27 cell line, derived from a tongue SCC patient, was a gift of Dr. Xinhong Wang (Medical college of Guangzhou). The UM-SCC-23 cell line was obtained from University of Michigan (a gift of Dr. Thomas E. Carey). Cell lines were authenticated by Shanghai XP Biomed Ltd. (China). Cal-27 cells and UM-SCC-23 cells were cultured in DMEM medium. HEK-293E cells and HeLa cells were maintained in RPMI medium.

### shRNAs, plasmids, and transfection

Mimics and inhibitors of five miRNAs (miR-503-5p, miR-450b-5p, miR-27a-3p, miR-181a-5p, and miR-183-5p) and negative control (NC) vector were synthesized by Suzhou Ribo Life Science Co. Ltd. (Suzhou, China). HeLa Cells were transfected with corresponding siRNA inhibitors or mimics diluted using Lipofectamine 2000 (Invitrogen) according to the manufacturer’s protocol.

For RORα knockdown, the lentivirus expression vectors GV-248-RORα-sh1, 2, 3 and GV-248 were purchased from Genechem and were verified by sequencing (Table S7).

For RORα overexpression, the lentivirus expression vector PCDH-EF1-puro was purchased from System Biosciences Company. We amplified the RORA transcript variant 1 (NM_134261.2) CDS sequence and inserted it into the PCDH-EF1-puro plasmid. The RORA CDS sequence was verified by sequencing.

### Luciferase reporter assay

The 3′ UTR sequence of RORA transcript variant 1 was amplified with primers (Table S8) to incorporate Xho I and Not I restriction sites. The PCR product was inserted into the pSiCheck™-2 dual luciferase reporter vector (Promega), and was verified by sequencing. For the luciferase reporter assays, wild type vectors or RORA-3′ UTR vectors were transiently co-transfected with miRNA mimics, inhibitors or NC vector into HeLa cells. Luciferase activity was measured 48 h post-transfection using Dual-Luciferase® Reporter Assay System (Promega). Renilla luciferase activity was normalized with firefly luciferase activity. The final normalized luciferase activity was normalized by 3′ UTR/NC control.

### Quantitative reverse-transcription PCR

Total RNA was isolated using the HP total RNA isolation kit (Omega bio-tek) following manufacturer’s instructions. 1 μg of total RNA was reverse-transcribed into cDNA using Takara®RT reagent Kit (Takara), and 0.8 μg of total RNA was used for miRNA reverse transcription with the Mir-X miRNA First-Strand Synthesis Kit (Takara). Quantitative PCR was performed using Roche FastStart Essential DNA Green Master (Roche) with the primers listed in Supplemental Table S8. The relative gene expression was calculated using the equation 2^-Δ(ΔCT)^ where ΔCt = Ct (mRNA)−Ct (actin) or Ct (miRNA)−Ct (U6).

### Immunohistochemical staining

Paraffinembedded specimens were sliced into 4 μm sections. After deparaffinating, dehydrating and performing antigen retrieval with high pressure, to quench the endogenous peroxidase activity, the sections were incubated with 3% hydrogen superoxide for 20 min followed by blocking non-specific binding in 10% normal goat serum. Immunohistochemical staining was performed as follows: The sections were incubated overnight at 4 °C with polyclonal rabbit anti-human RORα (1:50; Abcam, Cambridge, UK), then labeled with HRP secondary antibody (Universal streptavidin peroxidase kit including endogenous peroxidase blocking agent, goat serum, biotin-labeled goat anti-rabbit IgG, and horseradish peroxidase-labeled streptavidin; Zhongshan, Beijing, China) followed by DAB (Maxim, Fuzhou, Fujian, China) color reaction. The sections were then counterstained with haematoxylin.

### Scoring system and data visualization

The tissue microarray slices stained with RORα antibody were scanned by Aperio ScanScope CS scanner (Vista, CA, USA). Using Aperio ImageScope (Version 11.2), four fields (viewed at a magnification of ×100) of interests were selected in each spot. The nuclear positive ratio was calculated by counting the positive nuclear staining cells from total cancer cells and calculating the percentage. The intensity of staining was on the following scale: 0, no staining; 1+, mild staining; 2+, moderate staining; 3+, intense staining. The histoscore of nuclear staining for one field was calculated using the formula: score of one selected field = the nuclear positive ratio × the intensity score. The total staining score = field 1 + field 2 + field 3 + field 4.

### Protein extraction and western blotting

Total proteins were extracted using RIPA buffer supplemented with 1 mM PMSF, and concentration was measured by BCA protein assay kit (Thermo Scientific). Equal quantities of proteins were subjected to 10% SDS-PAGE and then electrophoretically transferred onto PVDF membranes. After blocking with 5% non-fat milk at room temperature for 1.5 h, the membranes were probed with primary antibodies against RORα (1:500, Abcam), p53(Ser46) (1:1000, Cell Signaling Technology), p53 (1:1000, Santa Cruz Biotechnology) or β-actin (1: 3000, Santa Cruz Biotechnology) overnight at 4 °C. Then, the membranes were incubated with horseradish peroxidase-conjugated secondary antibody (Santa Cruz Biotechnology) for 1 h at room temperature. The immunoreactive proteins were detected by ECL chemiluminescence system (Advanstar) and the β-actin was set as the normalized control.

### Cell counting kit-8 (CCK-8) assay

To assess the cell proliferation and viability, CCK-8 assay using a commercial kit (Dojindo, Tokyo, Japan) was performed following manufacturer’s instructions. Cells were seeded in 96-well plate in quintuplicate at a density of 5.0 × 10^3^ cells/well. The complete mediums inside wells were removed and replaced with corresponding complete mediums mixed with 10% CCK-8 solutions at different time points. After 2 h incubation at 37 °C, the absorbance value of each well was measured by a microplate reader.

### Mice xenografts

Cal-27 cells infected with PCDH-RORα or PCDH-puro were used for mice xenografts assay. 6–8 weeks old female BALB/c nude mice were randomly assigned to control or RORα-overexpressing group (*n* = 5) and were subcutaneously injected with 1 × 10^7^ cells in the flank region. One week after injection, the tumor growth was monitored every two days by measuring the length (the biggest diameter) and width (the smallest diameter) of tumors. The volume of tumor was calculated following the formula: volume = length × width^2^/2. The mice were sacrificed 30 days after injection, and the tumors were isolated, measured and weighed. All procedures were approved by the animal ethics committee of Wuhan University and performed following the guidelines of the Care and Use of Laboratory Animals (Ministry of Science and Technology of China, 2006).

### Statistical analysis

All quantitative experiments were performed in triplicate. GraphPad Prism 5.0 software system was used for data analyses and graphing. All data were presented as the mean ± SEM. A two-way ANOVA was used in CCK-8 assay and mice xenografts assay for group analysis. The patients’ survival analysis was obtained by Kaplan-Meier method, and the difference was determined by log-rank test. All other analyses were carried out using the student’s *t*-test (Two-tailed) to assess the statistical difference between groups. For all tests, *p* value < 0.05 were considered statistically significant.

## Electronic supplementary material

Supplementary materials summary

Supplemental Figure 1

Supplemental Figure 2

Supplemental tables

## Data Availability

All RNA-seq and miRNA-seq data supporting this article are accessible through NCBI’s gene Expression Omnibus accession number GSE107445.
