# Supplementary material for: MicroRNA-transcription factor network analysis reveals miRNAs cooperatively suppress RORA in oral squamous cell carcinoma
Source: Oncogenesis. 2018 Oct 8;7(10):79. doi: 10.1038/s41389-018-0089-8 (PMC6174157; doi:10.1038/s41389-018-0089-8)
Supplement: Supplementary file 1 — Supplementary materials summary [file 41389_2018_89_MOESM1_ESM.docx]

**Figure S1:** The size distribution of miRNA in human.

**Figure S2:** The predicted regulatory relationship between down-expressed miRNAs and targeted up-regulated mRNAs.

**Table S1:** Mapping of RNA-seq reads to reference genome.

**Table S2:** Expressed genes in each sample.

**Table S3:** Data obtained from miRNA-sequencing.

**Table S4:** Expressed miRNAs in each sample.

**Table S5:** The RPKM values of RORα isoforms in each samples from high-throughput sequencing.

**Table S6:** Patients clinical information.

**Table S7:** The sequences of RORα-sh1, 2, 3.

**Table S8:** Primers sets, related to the experimental procedures.
