# Supplementary figures and images for: MicroRNA-transcription factor network analysis reveals miRNAs cooperatively suppress RORA in oral squamous cell carcinoma
Source: Oncogenesis. 2018 Oct 8;7(10):79. doi: 10.1038/s41389-018-0089-8 (PMC6174157; doi:10.1038/s41389-018-0089-8)

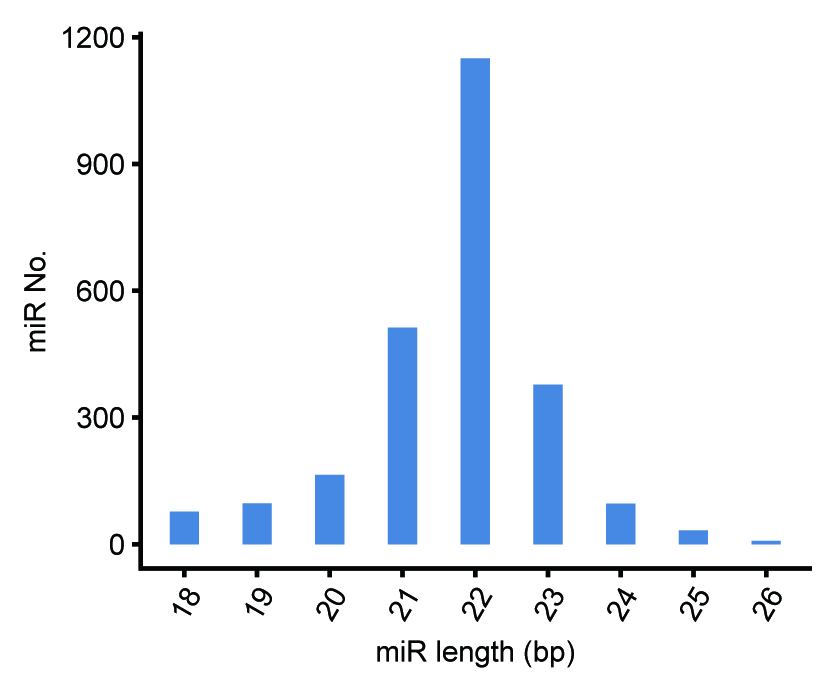

Supplement: Supplementary file 2 — Supplemental Figure 1 [file 41389_2018_89_MOESM2_ESM.tif]

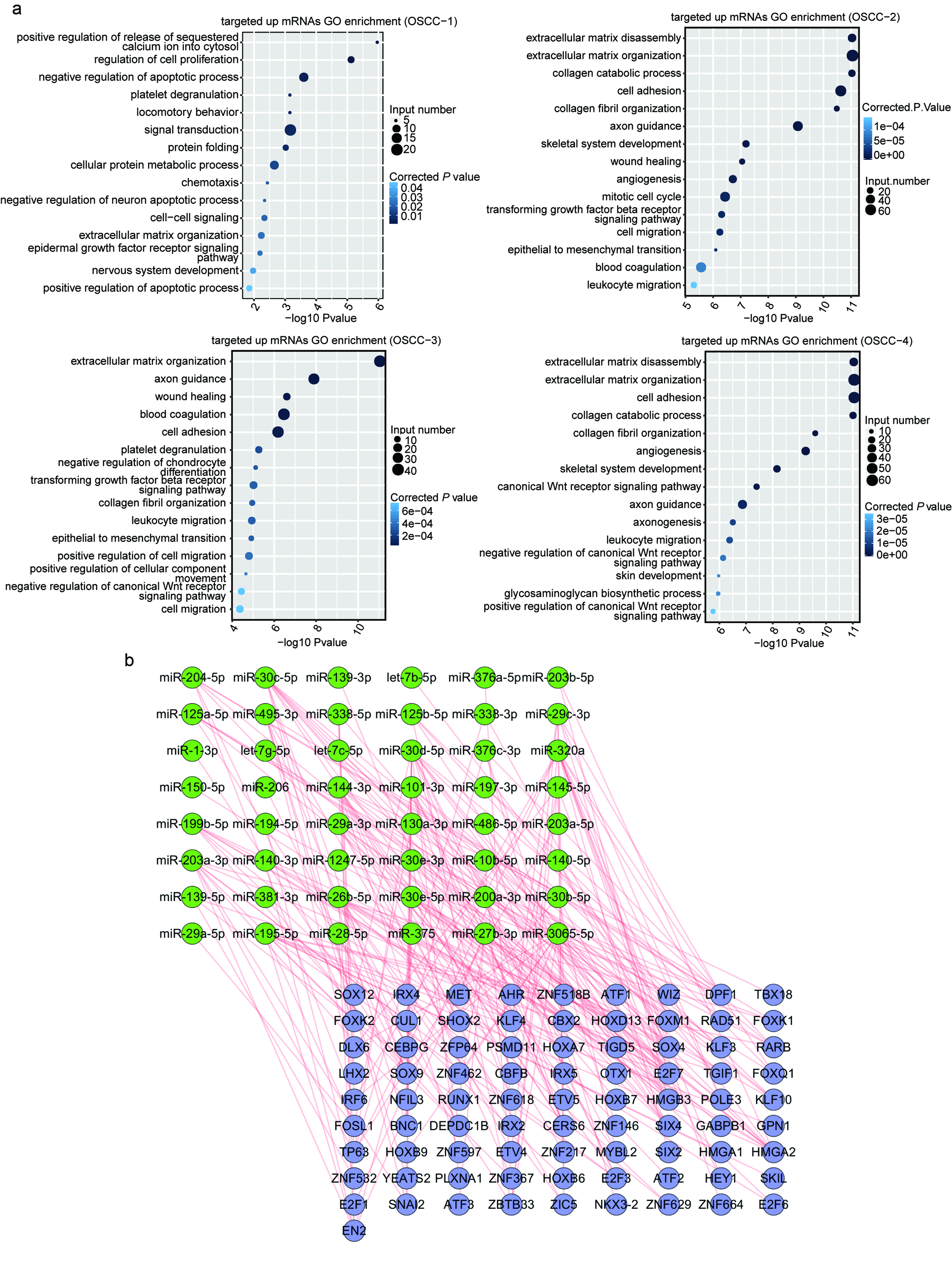

Supplement: Supplementary file 3 — Supplemental Figure 2 [file 41389_2018_89_MOESM3_ESM.tif]
